# Polyphenolic and Methylxanthine Bioaccessibility of Cocoa Bean Shell Functional Biscuits: Metabolomics Approach and Intestinal Permeability through Caco-2 Cell Models

**DOI:** 10.3390/antiox9111164

**Published:** 2020-11-22

**Authors:** Olga Rojo-Poveda, Letricia Barbosa-Pereira, Charaf El Khattabi, Estelle N.H. Youl, Marta Bertolino, Cédric Delporte, Stéphanie Pochet, Caroline Stévigny

**Affiliations:** 1 RD3 Department-Unit of Pharmacognosy, Bioanalysis and Drug Discovery, Faculty of Pharmacy, Université Libre de Bruxelles, 1050 Brussels, Belgium; Cedric.Delporte@ulb.be (C.D.); Caroline.Stevigny@ulb.be (C.S.); 2 Department of Agriculture, Forestry and Food Sciences (DISAFA), University of Turin, 10095 Grugliasco, Italy; letricia.barbosa.pereira@usc.es (L.B.-P.); marta.bertolino@unito.it (M.B.); 3 Department of Analytical Chemistry, Nutrition and Food Science, Faculty of Pharmacy, University of Santiago de Compostela, 15782 Santiago de Compostela, Spain; 4 Laboratory of Pharmacology, Pharmacotherapy and Pharmaceutical Care, Université Libre de Bruxelles, 1050 Brussels, Belgium; Charaf.El.Khattabi@ulb.ac.be (C.E.K.); yestella@yahoo.fr (E.N.H.Y.); Stephanie.Pochet@ulb.ac.be (S.P.); 5 Laboratory of Drug Development, Faculty of Medicine and Pharmacy, Université Joseph Ki-Zerbo, BP 958 Ouagadougou 09, Burkina Faso; 6 Analytical Platform of the Faculty of Pharmacy (APFP), Faculty of Pharmacy, Université Libre de Bruxelles, 1050 Brussels, Belgium

**Keywords:** cocoa bean shell, biscuits, functional foods, polyphenols, methylxanthines, in vitro digestion, α-glucosidase inhibition, bioaccessibility, metabolomics, Caco-2 absorption

## Abstract

Cocoa bean shell (CBS), a by-product with considerable concentrations of bioactive compounds and proven biofunctional potential, has been demonstrated to be a suitable ingredient for high-fiber functional biscuits adapted to diabetic consumers. In this work, the in vitro bioaccessibility and intestinal absorption of polyphenols and methylxanthines contained in these biscuits were evaluated, and the effect of the food matrix was studied. Biscuits containing CBS and the CBS alone underwent in vitro digestion followed by an intestinal permeability study. The results confirmed that compounds were less bioavailable in the presence of a food matrix, although the digestion contributed to their release from this matrix, increasing the concentrations available at the intestinal level and making them capable of promoting antioxidant and antidiabetic activities. After digestion, CBS biscuits were shown to possess α-glucosidase inhibition capacity comparable to that of acarbose. Moreover, the presence of the food matrix improved the stability of polyphenols throughout the digestion process. Intestinal absorption of flavan-3-ols seemed to be limited to a maximum threshold and was therefore independent of the sample, while procyanidin was not absorbed. Methylxanthine absorption was high and was boosted by the presence of the food matrix. The results confirmed the biofunctional potential of CBS-based biscuits.

## 1. Introduction

In recent years, functional foods and ingredients have received increasing interest. It has been claimed that when consumed in the diet, these foods possess the ability to treat, prevent, or reduce the risk of several pathologies, such as cancer, cardiovascular diseases, autoimmune disorders, diabetes, or even the process of aging through the neutralization of reactive oxygen species in the human organism. Polyphenols are one of the main compound groups responsible for the activity of different functional ingredients. These are secondary metabolites found in plants which have been demonstrated to have several health benefits in organisms, such as antioxidant, antidiabetic, and anticarcinogenic activities, among others [1]. In particular, great attention has been given to biofunctional compounds coming from food waste or food by-products as their use in foods would reduce the economic and environmental costs of their disposal, giving them a role in the sustainability and bioeconomical framework of the modern food industry [2]. In the cocoa industry, one of the main by-products is the cocoa bean shell (CBS). This cocoa by-product constitutes around 10–17% of the total cocoa bean weight, and more than 700 thousand tons are produced yearly worldwide. However, CBS contains several nutrients and non-nutritive microconstituents such as proteins, vitamins and minerals, dietary fiber, methylxanthines such as theobromine and caffeine, and polyphenols (mainly flavan-3-ols); thus, they have attracted interest from several researchers [3]. Moreover, it has been found that CBS presents a very similar aromatic profile to that of cocoa powder, which makes it even more attractive for functional food development [4,5]. Polyphenols contained in cocoa and CBS have been demonstrated to have several beneficial effects on the human organism, such as antimicrobial, antiviral, anti-inflammatory, anticarcinogenic, and antidiabetic effects, among others [3]. These effects are largely due to the antioxidative characteristics of this compound group, which cause them to act as chemopreventors of several chronic diseases [6]. Nevertheless, cocoa and CBS polyphenols could also promote beneficial effects such as antidiabetic actions by acting as enzyme inhibitors or modulating insulin secretion in pancreatic cells [7]. However, most of these bioactivities have been demonstrated in vitro through direct use of CBS extracts, without taking into account how the bioactive compounds contained in the cocoa by-product would change or what their availability would be during gastrointestinal digestion. The consumed compounds do not all arrive at the target organ in their original form or concentration to exert their biofunction, and sometimes, they may not even be released into the bloodstream [8].

The bioaccessibility of polyphenols during gastrointestinal digestion and their interactions with the food matrix are crucial factors that influence their bioavailability in the human organism and their actions at a local level within the gastrointestinal tract. This factor determines how much of the biofunctional compound fractions actually reach the intestinal tract and are available for absorption and passage into the bloodstream or action at the local level. It depends on different factors, such as compound release from the food matrix and the characteristics of this matrix, their solubilization degree, their molecular size, interactions with food components, hydrophilic and hydrophobic characteristics, or different types of transformation dependent on the pH that could take place [8,9].

Several studies have been carried out to develop functional foods containing CBS in order to revalorize this by-product. Due to its similarity to cocoa powder and its high fiber content, CBS has mainly been used to produce high-fiber baking goods with the ability to provide health benefits while utilizing a low-cost food ingredient [3]. However, little research has been carried out on the bioaccessibility of compounds of interest contained in these CBS functional foods or on how the food matrix could affect this parameter.

In this work, high-fiber CBS-based biscuits containing either sucrose or tagatose (a prebiotic sugar with low glycemic index adapted for diabetic consumers [10]) were studied to investigate the bioaccessibility and bioactivity (antioxidant and α-glucosidase inhibition capacities) of their bioactive compounds (polyphenol content and methylxanthines) after simulation of gastrointestinal digestion. Furthermore, the intestinal permeability of some targeted compounds was assessed. The effects of the biscuit food matrix on both bioaccessibility and intestinal permeability were evaluated. This work has a different approach and is a continuation of our previous work, in which technological aspects linked to the production of the biscuits were assessed [11].

## 2. Materials and Methods

### 2.1. Chemicals

Hexane (≥99%), acetone (≥99.5%), formic acid (≥98%), α-amylase from human saliva, pepsin from porcine gastric mucosa, bile salts, pancreatin from porcine pancreas, 4-(2-hydroxyethyl)-1-piperazineethanesulfonic acid (HEPES buffer; ≥99.5%), dimethyl sulfoxide (DMSO; 99.9%), Lucifer Yellow CH dipotassium salt, Folin and Ciocalteu’s phenol reagent, sodium carbonate (≥99.5%), sodium nitrite (≥99%), aluminum chloride (99%), (+)-catechin hydrate (>98%), methanol (≥99.9%), vanillin (99%), hydrochloric acid (37%), 2,2′-diphenyl-1-picrylhydrazyl (DPPH; 95%), 6-hydroxy-2,5,7,8-tetramethylchroman-2-carboxylic acid (trolox; 97%), α-glucosidase from intestinal acetone powder from rats, p-nitrophenyl-α-D-glucopyranoside (p-PNG; ≥99%;), potassium phosphate monobasic, acarbose (≥95%), epicatechin (>98%), caffeine (≥99%), MS quality formic acid, and MS quality methanol were obtained from Sigma-Aldrich (Milan, Italy; Steinheim, Germany). Theobromine (99%) was provided by Alfa Aesar (Kandel, Germany). Procyanidin B1 (PCB1; ≥99%) was obtained from Cayman Chemical Company (Ann Arbor, MI, USA). Calcium chloride dehydrate, hydrochloric acid, potassium chloride, potassium phosphate dibasic, sodium carbonate, sodium chloride, magnesium chloride hexahydrate, and ammonium carbonate were acquired from Carlo Erba (Milan, Italy). Sodium hydroxide (1M), ethanol (≥99.9%), and gallic acid were supplied by Fluka (Milan, Italy). D-(+)-Glucose was provided by Carl Both (Kalrlsruhe, Germany), and 3-(4,5-dimethylthiazol-2-yl)-2,5-diphenyltetrazolium bromide (MTT, 98%) was acquired from Acros Organics (Geel, Belgium). Minimum essential medium (MEM), Hank’s balanced salt solution (HBSS), fetal bovine serum (FBS), L-glutamine (200 mM), penicillin–streptomycin (10,000 U/mL), and sodium pyruvate (100 mM) were provided by Life Technologies (Paisley, UK).

Ultrapure water was prepared using a Milli-Q filter system (Millipore, Milan, Italy).

### 2.2. Samples and Formulation of the Model Food (Biscuits)

Roasted CBS extracted from Trinitario variety cocoa beans (São Tomé origin) was kindly supplied by Pastiglie Leone S.r.l. (Turin, Italy) and employed for the preparation of biscuits. CBS was micronized before model food preparation to obtain particles smaller than 20μm, as measured with a PSA 990 particle size analyzer (Anton-Paar GmbH, Graz, Austria).

Six biscuit types were prepared following the official method AACC 10-53.01 with slight modifications, as described by Rojo-Poveda et al. [11]. Two biscuit groups containing different types of sugar were developed, one with sucrose and one with the low-glycemic sugar tagatose. For each sugar group, a control biscuit was developed with no CBS added and another two types of biscuit were produced in which 10% and 20% of the wheat flour content was replaced with CBS flour (5.28% and 10.56% *w*/*w* for the final product). The obtained model biscuits were coded according to these characteristics: 0S (sucrose control biscuit with 0% CBS), 0T (tagatose control biscuit with 0% CBS), 10S (10% CBS and sucrose), 10T (10% CBS and tagatose), 20S (20% CBS and sucrose), and 20T (20% CBS and tagatose). For comparison purposes, and in order to study the food matrix effects, samples in which only CBS flour was present were prepared. These samples contained CBS quantities equivalent to those in 10% (CBS10) and 20% CBS (CBS20) biscuits, and water replaced the rest of the food matrix weight.

### 2.3. Preparation of Polyphenolic Extracts, In Vitro Digests, and Non-Digested Extracts

The biscuit and CBS ingredient alone samples were subjected to three different types of extraction: polyphenolic extraction with organic solvents (Org), simulated in vitro gastrointestinal digestion (Dig), and aqueous extraction (No_Dig).

For the polyphenolic extraction with organic solvents, 1 g of the sample was extracted following the procedure described by Mota-Gutierrez and co-workers [12]. Briefly, samples were washed with hexane for defatting, and the precipitate was dried and extracted by means of an acetone–water–formic acid solution (70:29.5:0.5). After centrifugation and filtration through 0.45 μm PTFE filters (LLG-Labware, CA, USA), the extract was evaporated and resuspended in 5 mL of ultrapure water.

Five grams of each sample underwent in vitro gastrointestinal digestion. The simulated digestion was performed following the three-stage model (oral, gastric, and intestinal phases) proposed by the INFOGEST consortium and described by Minekus et al. [13] with the slight modifications described in [14]. Briefly, during the oral phase (2 min), samples were mixed at 37 °C in a SW-20 water bath (Julabo GmbH, Seelbach, Germany) with an electrolyte solution to simulate salivary fluid and with amylase enzyme. Samples then underwent a gastric phase (2 h) in which they were mixed with simulated gastric fluid and pepsin enzyme. Finally, during the intestinal phase (2 h), samples from the gastric phase were mixed with simulated intestinal fluid, bile salts, and pancreatin. Solutions were adjusted to pH 3 and 7 for the gastric and intestinal phases, respectively, with a MICROpH 2002 pH-meter (CRISON, Carpi, Italy), and the digestion process was stopped by bringing the pH down to 5.4. Samples were then centrifuged at 12,500× *g* and 0 °C for 10 min in a Heraeus megafuge 11R centrifuge (THERMO electron, Chateau-Gontier (France), and the supernatants were filtered through a 0.45 μm CA syringe filters and immediately stored at −20 °C until further analysis. The digestion process was replicated four times for each sample. Digestion blanks in which the whole sample was replaced with 5 mL of water were also prepared in order to monitor the possible contributions of the enzymes and salts employed during in vitro digestion in the subsequent analytic determinations.

Non-digestion extraction was performed to allow a comparison with the samples digested in vitro. This employed the same temperature and time conditions and the same weight and volume ratios. Samples were extracted at 37 °C for 4 h and 2 min (the whole time for the simulated gastrointestinal digestion) with just Milli-Q water and without enzymes or simulated fluids in order to avoid food matrix degradation due to those components. After this, non-digested samples were centrifuged and filtered in the same way as the digested samples.

### 2.4. Analytical Determinations

#### 2.4.1. Total Phenolic, Flavonoid, and Tannin Contents

The total phenolic content (TPC), total flavonoid content (TFC), and total tannin content (TTC) were assessed by the Folin–Ciocalteu, aluminum chloride, and vanillin methods, respectively. Methods were adapted as described in [15] for analysis in 96-well microplates, and a BioTek Synergy HT spectrophotometric multi-detection microplate reader (BioTek Instruments, Milan, Italy) was employed. For TPC determination, a gallic acid standard curve was prepared (20–100 mg/L), and the results are expressed as milligrams of gallic acid equivalents (GAE) per gram of sample. For TFC and TPC, quantification was performed by using a calibration curve of catechin (5–500 mg/L), and the results are expressed as milligrams of catechin equivalents (CE) per gram of sample. All measurements were performed in triplicate for all of the different sample extracts (Org, Dig, and No_Dig), and the values obtained for the control biscuits S0 and T0 were used as blanks and subtracted from S10 and S20 and T10 and T20, respectively, in order to adjust the final results and avoid food matrix interference, as indicated in [16].

#### 2.4.2. Radical Scavenging Activity

The radical scavenging activity (RSA) or antioxidant capacity of the samples was determined by the DPPH radical-scavenging method described by [15] in 96-well plates using the BioTek Synergy HT spectrophotometric reader (BioTek Instruments). The RSA was calculated as the inhibition percentage (IP) of the DPPH radical according to the following equation:

$$\mathrm{IP}\;\left(\%\right)=\;\left(A_{0}-A_{30}\right)/A_{0}\times 100$$

where A_0_ is the absorbance at the initial time, and A_30_ is the final absorbance after 30 min.

A calibration curve of Trolox was employed (12.5–300 μM) for the quantification of radical scavenging activity, and the results are expressed as micromoles of Trolox equivalents (TE) per gram of sample. All measurements were performed in triplicate for all of the different sample extracts (Org, Dig, and No_Dig), and the values obtained for the control biscuits S0 and T0 were used as blanks and subtracted from S10 and S20 and T10 and T20, respectively, in order to avoid food matrix interference.

#### 2.4.3. In Vitro α-Glucosidase Inhibition Capacity

The α-glucosidase inhibition capacity of the samples was determined by the α-glucosidase colorimetric assay described in [17] by employing 96-well plates and reading results with the BioTek Synergy HT spectrophotometric reader (BioTek Instruments). The enzyme inhibition capacity of the samples was calculated as their inhibition percentage and expressed as micromoles of acarbose equivalents (AcE) per gram of sample by using an acarbose calibration curve (1–25,000 μM). All measurements were performed in triplicate for all of the different sample extracts (Org, Dig, and No_Dig) and the values obtained for the control biscuits S0 and T0 were used as blanks and subtracted from S10 and S20 and T10 and T20, respectively, in order to avoid food matrix interference.

#### 2.4.4. Liquid Chromatography–High-Resolution Mass Spectrometry (LC–HRMS) Analysis

Characterization of polyphenols and methylxanthines from all the extract types (Org, Dig, and No_Dig) of the samples was performed with an untargeted metabolomics approach by using a rapid-resolution LC system (RRLC) 1200 series (Agilent Technologies, Waldbronn, Germany), including a binary pump, thermostatized autosampler, column oven, and a DAD detector. The RRLC system was coupled to a 6520 series electrospray ionization (ESI) source with a quadrupole time-of-flight (QTOF) mass spectrometer (Agilent Technologies). Compound separation was performed using an InfinityLab Poroshell 120EC-C18 column (2.1 × 100 mm, particle size of 2.7 μm) with a guard column (2.1 × 5 mm) (Agilent Technologies). The column temperature was kept at 35 °C. Water acidified with 0.1% formic acid (solvent A) and methanol with 0.1% formic acid (solvent B) served as mobile phases. A gradient elution at a flow rate of 0.45 mL/min was conducted as follows: minutes 0–1, 99% A and 1% B; minutes 1–15, linear gradient from 1% to 80% B, minutes 15–17, 80% B; minutes 17–17.5, linear gradient from 80% to 90% B; minutes 17.5–20, 90% B; minutes 20–21, linear gradient from 90% to 1% B; minutes 21–28, 1% B. The sample injection volume was 5 μL. The ESI–QTOF settings for metabolomics analysis were as follows: positive mode, 2 GHz resolution, MS scan range and rate of 100–1500 *m/z* at 1 spectra/s, drying gas temperature of 325 °C, drying gas flow of 10 L/min, nebulizer pressure of 45 psi, capillary voltage of 4000 V, and fragmentor voltage of 150 V. Reference ions of *m/z* 121.050873 and 922.009798 were infused and used as continuous ions for mass calibration during analysis. Nitrogen was used as a nebulizer and drying gas. Data acquisition and analysis were carried out by MassHunter Acquisition^®^ software for QTOF (Version B.04 SP3) and MassHunter Qualitative Analysis^®^ (Version B.08) software (Agilent Technologies). Samples were randomly analyzed in one single batch. The same quality control (QC = pool) sample for all samples (mix of all samples) was injected throughout the run after approximately every ten samples as a control, and blanks (water) were also injected throughout the run.

For the samples from the permeability study through Caco-2 cells, the quantification of five targeted compounds identified by MS was performed using calibration curves of caffeine, theobromine, epicatechin, catechin, and procyanidin B1 (PCB1) standards drawn from 0.01 to 10 ppm. LC-HRMS analysis was performed as mentioned above with slight modifications: 20 μL of sample was injected and analyses were performed in positive (for methylxanthines) and negative (for epicatechin, catechin and PCB1) modes. MassHunter Quantitative Analysis^®^ (Version B.08) software (Agilent Technologies) was employed for concentration determination of the samples.

LC–HRMS Data Processing and Statistical Analysis (Metabolomics Study)

Agilent format d data were converted to.mzXML format using the Peak Picking filter option in ProteoWizard MSConvert tools software (Version 3.03.9393, 64-bit). Preprocessing (filtration, peak identification, peak grouping and smoothing, retention time correction, integration, and annotation), normalization, quality control (metabolites correlation analysis and determination of batch correction), and statistical analysis (multivariate modeling and univariate testing) were conducted using the Galaxy workflow4metabolomics W4M online and freely available platform (http://workflow4metabolomics.usegalaxy.fr [18], version 3.3). The parameters used for processing and statistics were based on a previous study [19], with slight modifications, and can be viewed in Table S1 of the Supplementary Material accompanying this article.

### 2.5. Cell Culture and Assays

Human colon adenocarcinoma Caco-2 cell lines obtained from the American Type Culture Collection (ATCC, Rockville, MD, USA) were used between passages 10 and 42 for cell viability assays and permeability studies with the digested extracts. Cells were cultured in MEM supplemented with L-glutamine (2 mM), penicillin–streptomycin (100 U/mL–100 μg/mL), pyruvate (1 mM), and fetal bovine serum (10% *v/v*) in a 5% CO_2_ atmosphere at 37 °C inside a Binder incubator (Tullingen, Germany).

#### 2.5.1. Cell Viability Assay

The absence of cytotoxicity from digested extracts was evaluated by MTT assay. Caco-2 cells were seeded into 96-well plates at a density of around 20,000 cells/well and left to grow for 48 h at 37 °C and 5% CO_2_. After incubation, culture medium was removed by aspiration, replaced by 100 μL of digested extract (1/4, diluted in culture medium), and left for incubation for either 2 or 4 h. Then, the extracts were removed, and 100 μL of MTT (0.5 mg/mL) was added. After 3 h of incubation in the dark in a humidified atmosphere (5% CO_2_, 37 °C), plates were centrifuged at 560× *g* for 20 min, the supernatant was removed by returning the plate, 100 μL of DMSO was added to each well, and the plates were shaken for 5 min in order to dissolve the reduced intracellular formazan created. The absorbance was then read at 540 nm in a BioTek Synergy HT spectrophotometric multi-detection microplate reader (BioTek Instruments, Winooski, VT, USA). The percentage of cell viability was assessed in relation to the value given by the culture medium control, and values higher than 80% were considered acceptable. Experiments were performed in quadruplicate with at least five replicates.

#### 2.5.2. Permeability Study through Caco-2 Cell Monolayers

Permeability assays were performed following the protocol described by Hubatsch et al. [20] with slight modifications. Caco-2 cells were seeded into the apical side (AP) of hanging inserts with a pore size of 0.4 μm and a diameter of 1.12 cm placed on 12-well plates (Greiner Bio-One, Vilvoorde, Belgium). Culture medium (0.5 mL) containing approximately 300,000 cells was added in the apical side (AP) of each insert to give a final cell density of around 260,000 cells/cm^2^, and 1.5 mL of culture medium was added to the basolateral side (BL). Cells were left to grow for 21–29 days to differentiate and form a monolayer, with the medium being changed every 2–3 days. For this, aspiration was carried out in the BL and inserts were returned for the AP emptying in order to maintain the integrity of the monolayer. After this period, the transepithelial electrical resistance (TEER) of the monolayers was monitored using a STX2 chopstick electrode coupled to an EVOM2 epithelial voltmeter (World Precision Instruments, Sarasota, FL, USA), and TEER values were calculated in Ω·cm^2^ after subtraction of the blank value given by an empty insert without cells. Cells were considered to have reached confluence when TEER values were higher than 260 Ω·cm^2^ [20].

The culture medium was changed 24 h prior to the transport study day. Before the permeability assay, the culture medium was removed and 0.5 and 1.5 mL of modified HBSS (with 25 mM D-glucose, 20 mM HEPES, 1.25 mM CaCl_2_, and 0.5 mM MgCl_2_) were added to the AP and to the BL, respectively, and incubated for 30 min before TEER measurement (TEER values before the test). Modified HBSS was then removed and 0.4 mL of digested extract (diluted 1:4 with modified HBSS) was introduced into the AP, while 1.2 mL of modified HBSS was added to the BL. Plates were placed under agitation at 37 °C, and after 60 or 120 min (T1 and T2, respectively), 0.5 mL of the sample was taken from the BL, followed by the addition of the same quantity of modified HBSS. Samples were stored at −20 °C until further analysis by LC–HRMS. TEER measurement was carried out at the end of the assay in order to confirm the integrity of the monolayer by comparing it with the values obtained before analysis. Permeability analyses were performed in triplicate, and the apparent permeability coefficient (P_app_) was calculated as follows [21,22]:

$$P_{\mathrm{app}}=\mathrm{dC}/\mathrm{dt}\;\times V/(A\times C_{0})$$

where dC/dt is the change in the concentration of the compound on the basolateral side (receiving chamber) over time (or slope of the cumulative concentration), V is the volume in the basolateral chamber, A is the membrane surface area, and C_0_ is the compound’s initial concentration on the apical side (donor chamber).

#### 2.5.3. Study of the Caco-2 Cell Monolayer Integrity by Means of the Lucifer Yellow Assay

In addition to TEER measurements taken before and after the permeability assay, the integrity of the monolayer after the permeability test was also confirmed using Lucifer Yellow, which can serve as a marker for monolayer imperfections, since it is a molecule that is unable to permeate through lipophilic barriers. To do so, the employed inserts were emptied and introduced into a new plate. Then, 1 mL of modified HBSS was added into the BL, and 0.5 mL of Lucifer Yellow (0.1 mg/mL of modified HBSS) was added into the AP. Plates were left for incubation for 60 min at 37 °C without agitation. After this, quadruplicate 150 μL samples of the BL were placed into 96-well plates, and fluorescence was measured with excitation at 485 nm and emission at 530 nm. The percentage of Yellow Lucifer permeability (with a maximum acceptance of 3%) was calculated as follows:

$$\%\;\mathrm{permeabilty}\;\left(\mathrm{maximum}\;\mathrm{acceptable}=3\%\right)=\;\frac{\mathrm{sample}-{\mathrm{blank}}_{\mathrm{HBSS}}}{\mathrm{LY}-{\mathrm{blank}}_{\mathrm{HBSS}}}\times 100$$

where blank_HBSS_ is the fluorescence value given by a blank of modified HBSS, and LY is the fluorescence value given by a 0.1 mg/mL solution of Lucifer Yellow.

### 2.6. Statistical Analyses

The results obtained from spectrophotometric assays (TPC, TTC, TFC, radical scavenging activity, and α-glucosidase inhibition capacity) were analyzed through one-way analysis of variance (ANOVA) with Duncan’s post-hoc test at a confidence level of 95% in the 26.0.0.1 version of SPSS Statistics software (IBM-SPSS Inc., Chicago, IL, USA).

Data obtained from the LC–HRMS analysis for metabolomics study were subjected to univariate testing and multivariate modeling (principal component analysis, PCA; and partial least squares discriminant analysis, PLS-DA) using the above-mentioned W4M platform.

## 3. Results

### 3.1. Total Phenolic, Tannin, and Flavonoid Contents

**Figure 1:**
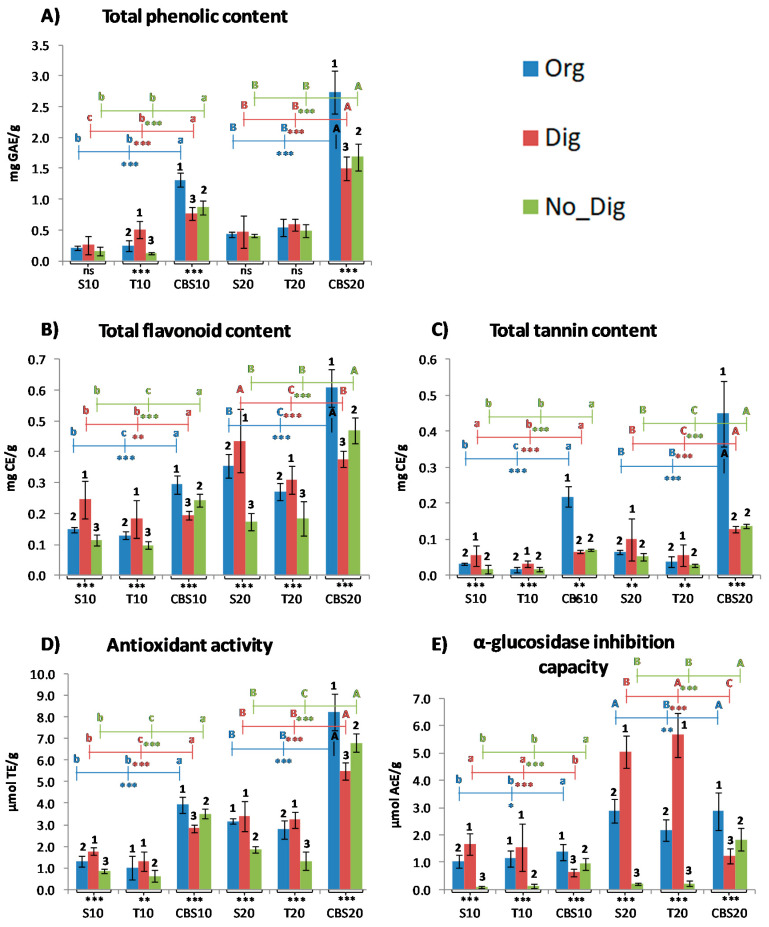
Total phenolic content (**A**), total flavonoid content (**B**), total tannin content (**C**), antioxidant capacity (**D**), and α-glucosidase inhibition capacity (**E**) for the three extraction types (Org, polyphenolic extraction with organic solvents; Dig, in vitro digestion; and No_Dig, no digestion) performed for the cocoa bean shell (CBS) biscuits (S10, T10, S20 and T20) and equivalent quantities of CBS powder alone without the food matrix (CBS10 and CBS20). The results are presented as the mean ± standard deviation (*n* = 6). Statistical comparisons were made using one-way ANOVA and Duncan’s post-hoc tests at 95% confidence. For each parameter, the different numbers above the bars indicate significant differences at *p* < 0.05 for the three different types of extraction within the same sample. Different letters indicate significant differences at *p* < 0.05 for the same extraction within samples with equivalent CBS percentage (lowercase = 10% CBS and uppercase = 20% CBS). Significance: * *p* < 0.05; ** *p* < 0.01; *** *p* < 0.001; ns = not significant. S10 (biscuit with sucrose and 10% of wheat flour substituted with CBS), T10 (biscuit with tagatose and 10% of wheat flour substituted with CBS), CBS10 (CBS powder alone without food matrix in equivalent quantity to that in 10% biscuits), S20 (biscuit with sucrose and 20% of wheat flour substituted with CBS), T20 (biscuit with tagatose and 20% of wheat flour substituted with CBS), and CBS20 (CBS powder alone without food matrix in equivalent quantity to that in 20% biscuits).

The results for the TPC, TFC, and TTC analyses performed for the three extraction types (organic extraction, in vitro gastrointestinal digestion, and no digestion) of all samples (biscuits containing CBS and samples with equivalent quantities of the CBS ingredient alone) are shown in Figure 1A–C, respectively, and their numerical values are available in Table S2.

Considering all of the different extractions, TPC values were between 0.13 and 0.50 mg GAE per gram of biscuit for the biscuits in which 10%of wheat flour was substituted by CBS, while the values for the equivalent quantity of CBS without the food matrix were 0.77–1.32 mg GAE/g. For the S20 and T20 biscuits, TPC values ranged from 0.41 to 0.59 mg GAE/g, while samples with the equivalent quantity of CBS alone gave TPC values of 1.50–2.73 mg GAE/g. These values are in accordance with those obtained by different researchers for other functional foods containing similar recovered ingredients, such as biscuits containing 4.2% coffee fiber from spent coffee grounds (up to 8.98 mg GAE/g of biscuit digest) [23], drinking yogurt containing CBS extract (1.31–2.01 mg GAE/mL) [24], and different CBS-based beverages (0.3–1.8 mg GAE/mL) [14,17].

Generally, no differences associated with the sugar employed for the biscuits were observed. However, significant differences were found when comparing the CBS biscuits with samples with an equivalent quantity of CBS without a food matrix. These differences might be justified by the fact that the CBS ingredient alone underwent a single roasting process—the one undergone by cocoa beans before separating them from the CBS during their manufacture—while the phenolic compounds contained in the biscuits may have undergone extra degradation due to the second high-temperature step during biscuit baking. It has already been demonstrated that a similar baking process to that used in this work could cause a loss in TPC value of up to 50% [16]. In addition, some losses in TPC and biofunctionality (RSA and α-glucosidase inhibition capacity) were expected for the biscuits in comparison with the CBS ingredient alone, since the phenolic contents responsible for these activities have been reported to easily link to the food matrix provided by the ingredients of the biscuits, losing some of their activity. Polyphenols can combine with other components of the food matrix by both hydrogen and covalent bonds. Mostly, they combine with the cell wall components that constitute dietary fiber [25,26]. Dietary fiber in the biscuits with zero CBS added (control biscuits) has been reported to account for 2.48–2.79% of the dried product [11].

TFC values were between 0.10 and 0.25 mg CE/g of biscuit for S10 and T10 and between 0.17 and 0.44 mg CE/g of biscuit for S20 and T20, while values for the CBS ingredient without a food matrix ranged from 0.19 to 0.29 mg CE/g for CBS10 and between 0.38 and 0.61 mg CE/g for CBS20. These values were all in accordance with those previously reported for other types of foods based on CBS, such as drinking yogurt (0.20 mg CE/mL) [24] and CBS beverages (0.01–0.55 mg CE/mL) [17]. In the case of flavonoids, the values obtained for the biscuits accounted for at least half of the TPC values, while values of the CBS ingredients alone were proportionally lower in comparison with the former. This could be due to higher stabilization of this kind of polyphenol in the presence of a food matrix, which would probably reduce its degradation during the biscuit baking process. TFC values corresponding to organic extraction and non-digestion were proportionally well correlated with those of TPC (*r* = 0.8985 and *r* = 0.9943, respectively) and followed a similar variation trend, while the TFC values of the sample digested in vitro showed a much lower correlation with the TPC results (*r* = 0.3044). Indeed, the TPC values obtained for the samples of CBS10 and CBS20 digested in vitro were almost double those of their biscuit counterparts, while the TFC values of the CBS ingredients alone were in line with those of their biscuit counterparts or were even significantly lower (e.g., TFC of digested CBS20 was lower than TFC S20). A hypothetic explanation for this could be that, for the biscuits, a higher concentration of flavonoids was present after gastrointestinal digestion thanks to the liberation of these compounds, which were previously linked to the food matrix. On the other hand, it is also possible that these values increased because of the degradation of polymeric flavonoids during digestion (e.g., condensed tannins) into their monomeric flavonoid compounds.

Values of TTC for the three extractions followed a similar pattern to TPC, where higher values were found for CBS10 and CBS20 (0.07–0.22 mg CE/g and 0.13–0.45 mg CE/g, respectively) than for their biscuit counterparts (0.02–0.05 mg CE/g for the 10%-substitution biscuits and 0.03–0.10 mg CE/g for the 20%-substitution biscuits). In the case of the tannin content, values accounted for 5.72–21.09% of the TPC, which is in accordance with the 3.4–19.2% previously found for beverages in a previous study using the same CBS powder [17]. Strong correlations of the TTC and TPC values were again found for the organic and non-digestion extractions (*r* = 0.9940 and *r* = 0.9789, respectively) and, contrary to the TFC, the TTC–TPC correlation for the samples digested in vitro was also high (*r* = 0.7288).

### 3.2. Biofunctional Characteristics—Radical Scavenging Activity and α-Glucosidase Inhibition Capacity

In this study, two parameters related to the biofunctional properties provided by CBS were studied in the biscuits and in the CBS ingredient alone: the radical scavenging activity (RSA) or antioxidant capacity, and the α-glucosidase inhibition capacity, which can, in part, contribute to the antidiabetic capacity of the products by inhibiting the enzyme involved in the degradation of glucose polymers into glucose, therefore decreasing glucose absorption and postprandial glycemia.

The radical scavenging activity of the CBS containing biscuits and the samples with an equivalent quantity of the CBS ingredient alone was determined for the three different extractions (polyphenolic organic extraction, in vitro gastrointestinal digestion, and no digestion). The results are presented in Figure 1D, and the numerical values are available in Table S2. RSA values were between 0.64 and 1.78 μmol TE/g of biscuit for S10 and T10, between 1.33 and 3.40 μmol TE/g of biscuit for S20 and T20, and between 2.83 and 3.93 and 5.49 and 8.23 μmol TE/g for the equivalent quantities of the CBS ingredient alone (CBS10 and CBS20, respectively). As reported previously in other works on CBS functional compound extraction [15], the RSA was highly correlated with TPC, TFC, and TTC, and varied proportionally to those. Again, the RSA results of our samples were in line with the values previously obtained for other functional foods developed with CBS, such as different CBS-based beverages (1–7.29 μmol TE/mL) [14,17], drinking yogurt with added CBS extract (up to 1.92 μmol TE/mL) [24], or functional cakes where CBS was used as a fat replacement (1–4.8 μmol TE/g) [27].

Values for the α-glucosidase inhibition capacity measured for all the samples and extractions are shown in Figure 1E, and their numerical values are available in Table S2. These results are expressed as micromoles of acarbose equivalents per gram (μmol AcE/g). Acarbose is an α-glucosidase inhibition drug that is used to treat diabetes by decreasing polysaccharide digestion in the digestive tract and, therefore, producing a decrease in glucose absorption and avoiding arise in the glycemic index. Values between 0.10 and 1.66 μmol AcE/g were found in the different extractions of both S10 and T10, and a similar range was obtained for the CBS ingredient alone (CBS10; 0.63–1.39 μmol AcE/g). For the 20% samples, however, higher values were found for the biscuits (0.20–5.65 μmol AcE/g biscuit for S20 and T20) in comparison with CBS20 (1.22–2.87 μmol AcE/g), contrary to what was observed for the other parameters measured previously. This is mainly due to the significantly higher levels of activity found for the digested biscuit samples, which suggests that significant liberation or transformation of the compounds responsible for the enzyme inhibition occurred during simulated gastrointestinal digestion. These values were, again, in line with the previously reported α-glucosidase inhibition capacities of different foods based on CBS or other biofunctional by-products, such as the coffee fiber cookies studied by Martinez-Saez et al. [23], for which up to 2.04 μmol AcE/g of digested biscuit was obtained, or the different CBS beverage and flavored CBS beverage preparation methods studied by Rojo-Poveda et al. [17] and Cantele et al. [14], which showed enzyme inhibition of up to 0.5 μmol AcE/mL. The obtained values are very interesting if we take into account that when consuming and digesting a CBS-containing biscuit (approximately 15 g), up to 84.75 μmol of AcE could be available and could act by inhibiting the α-glucosidase enzyme at the intestinal level. Considering this dose, an intake of a few biscuits would provide acarbose equivalent values very close to the daily dose of acarbose used for postprandial glycemic control (150–200 mg, which is equal to 232–310 μmol) [28]. In this way, it would be possible to obtain some of the desired enzyme inhibition through the diet, while avoiding the unpleasant side effects of acarbose, including gastrointestinal symptoms such as diarrhea, flatulence, abdominal discomfort, and even abnormal liver function in some rare cases [29].

### 3.3. In Vitro Bioaccessibility of Bioactive Compounds and Functional Characteristics through Spectrophotometric Analysis Results

For all parameters obtained through spectrophotometric assays (Figure 1), a general trend was repeated, probably because of the interdependence of these parameters. Generally, the No_Dig samples gave the lowest values when comparing them to the other extractions, while the Dig samples presented the highest values for the biscuit samples and the lowest for the CBS ingredient samples without a food matrix. In contrast, organic extraction (Org) led to the highest values for the CBS ingredient samples. Additionally, significant differences were generally found within the same CBS percentage between the biscuit and CBS ingredient samples, since the latter gave higher values than the biscuits in almost all cases. This was probably caused by the two reasons already explained in previous sections: the biscuits underwent an extra high-temperature process that the CBS ingredient alone samples did not, and some bonds between the food matrix of the biscuits (mostly fibers and proteins) and the bioactive compounds could have been created, preventing their liberation from the matrix, as was already reported by Hilary et al. [26] for date-seed-functionalized breads in contrast with date seed extract alone. It is worth mentioning that little or even no significant differences were found when comparing biscuits made with the same quantity of CBS but with different sugars, which suggests that the sugar type does not influence the bioaccessibility of the bioactive compounds at this level. Nevertheless, after observing these repeated differences, two effects were hypothesized: a compound liberation effect and a food matrix protection effect.

The compound liberation effect can be justified by the higher values found for digested CBS biscuits in comparison with their non-digested counterparts. Thus, it is suggested that the destruction of the food matrix during gastrointestinal digestion could lead to the liberation of polyphenolic compounds that were initially bound to this food matrix. This effect has been previously reported in other works for polyphenols that were included in a food matrix. Bertolino et al. [30] found a 12% increase in TPC and a 61% increase in RSA after the digestion of coffee silverskin yogurt, which was attributed the hydrolysis of polyphenolic compounds from polysaccharides and proteins by digestive enzymes. Barbosa-Pereira et al. observed slight increases in TFC and TTC and up to 50% and 25% increases in TPC and RSA, respectively, after simulated digestion of CBS-based ice cream [31]. Kan et al. [32] observed greater bioaccessibility after intestinal digestion of anthocyanins contained in blueberry-fortified breads, which was not observed when a non-fortified bread was codigested with the equivalent quantity of blueberry extract. Paz-Yépez et al. [33] found that the bioaccessibility of polyphenols contained in dark, milk, and white chocolate considerably increased after gastrointestinal digestion by up to several times the initial values for non-digested chocolate.

On the other hand, the opposite effect was found for the digested samples of the CBS ingredient alone, which led to the food matrix protection effect hypothesis. Indeed, when observing the different values obtained for CBS10 and CBS20, it was noted that the digested samples gave the significantly lowest values, followed by the non-digested samples and those that underwent organic extraction, which gave the significantly highest values in almost all cases for the five parameters measured through spectrophotometric techniques. This was probably due to the absence of the food matrix protection effect, which led to the degradation of polyphenolic compounds caused by the attack of digestive enzymes and acids and to pH changes during the in vitro simulated gastrointestinal digestion. This was illustrated by the work of Cantele et al. [14] in which the TPC, RSA, and α-glucosidase inhibition capacity values decreased after gastrointestinal digestion by up to 76%, 57%, and 52%, respectively, for CBS-based beverages, since no food matrix was present in these food samples to protect the polyphenolic compounds. Tarko et al. [34] demonstrated that polyphenols linked to the food matrix are less bioavailable, although they are less easily degraded during digestion when contained in matrices such as whole fruits than when found in extracts. Moreover, Pešić et al. [35] found that a meat- and cereal-based food matrix mixed with grape seed extract increased the total recovery of polyphenols after simulated digestion in comparison to the extracts digested without the presence of the food matrix. Indeed, several authors have stated that the fat content in food matrices can significantly increase polyphenol bioaccessibility and stability during gastrointestinal digestion by interacting with these bioactive compounds by micellarization [25,36], which would be justified in the case of the butter-containing biscuits presented in this work, in contrast to the samples of CBS ingredient alone. Ortega et al. [36] found that the higher fat content in cocoa liquor (approximately 50%) in comparison with cocoa powder (approximately 15% fat content) enhances the digestibility of some of its phenolic compounds thanks to a protective effect during digestion. This effect was also observed in another study in which the bioaccessibility of three different types of chocolate increased after gastrointestinal digestion and the greater the fat content was, the more the bioaccessibility percentage increased (dark chocolate < milk chocolate < white chocolate) [33].

### 3.4. LC–HRMS and Metabolomics Analysis for Single Compound Bioaccessibility Evaluation

The samples of biscuits with different sugars and different CBS percentages as well as the samples with an equivalent quantity of the CBS ingredient alone underwent three different types of extraction (Org, Dig, and No_Dig) and were analyzed through reverse-phase LC–HRMS. The W4M platform was used to conduct an untargeted metabolomics study on the obtained data, and a total of 1585 features were obtained after data processing and filtering, which were then subjected to multivariate and univariate analyses.

**Figure 2:**
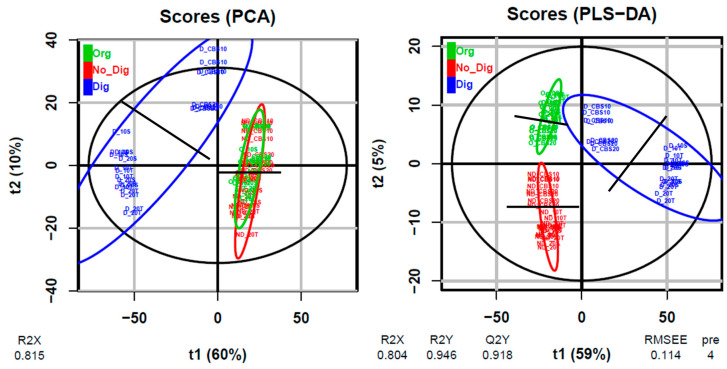
Score plots for the multivariate modeling of variations using PCA (principal component analysis) or PLS-DA (partial least squares discriminant analysis) of all samples (biscuits and equivalent quantities of CBS alone without a food matrix) which were subjected to three different types of extraction (Org, No_Dig, and Dig). For each plot, the percentages of total variation explained by components 1 and 2 (t1 and t2, respectively) are indicated in parentheses. Black ellipses include 95% of the multivariate normal distribution for all samples (subgrouped in colored ellipses according to the extraction type).

In order to show the general differences within the whole sample set, Figure 2 shows the PCA and PLS-DA plots obtained from the multivariate untargeted metabolomics study performed on all samples together. Supplementary information on these plots is available in Figure S1. Both plots allowed for clear separation of the digested samples from the other two extraction types. This separation was mainly driven by the horizontal axis, which corresponds to the first component t1, which explained 60% and 59% of the total variation in the PCA and PLS-DA plots, respectively. The observed separation may suggest and confirm the changes in polyphenolic compositions during the digestion process due to the attack of enzymes and acids. Additionally, PLS-DA also allowed for separation of the organic and non-digestion extractions. On the other hand, the second component, which explained 10% and 5% of the total variation in PCA and PLS-DA, respectively, may reflect another separation that was observed for the vertical axis in both plots. For all extraction types, this second separation allowed the CBS ingredient alone samples to be separated from the biscuit samples (separation indicated by black lines in each colored ellipse) and, in some cases, it was also possible to visualize the vertical separation of the 20%-substitution samples from the 10%-substitution samples. Thus, the second component was probably driven by the different polyphenol concentrations found in the samples. Again, no differences associated with the employed sweetener were observed between biscuits.

**Figure 3:**
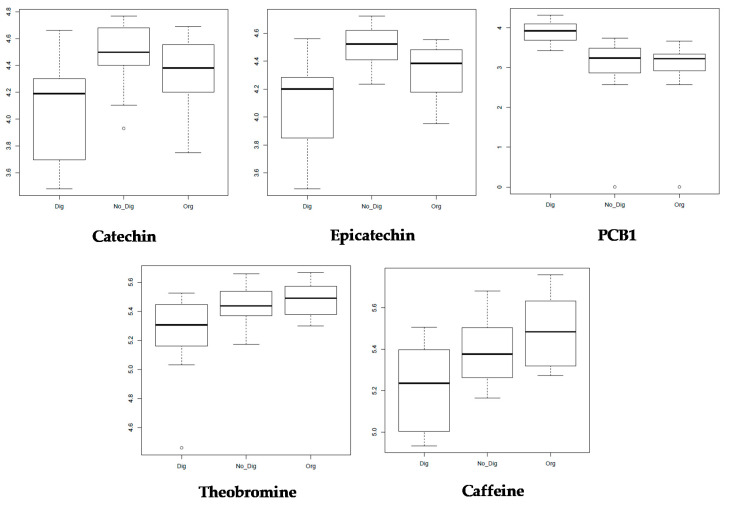
Boxplot of the log_10_-intensities of catechin (*m/z* = 291.1070, t = 497 s), epicatechin (*m/z* = 291.0969, t = 585 s), PCB1 (*m/z* = 579.2428, t = 378 s), theobromine (*m/z* = 181.3165, t = 371 s), and caffeine (*m/z* = 195.2854, t = 491 s) for the three types of extraction for the whole sample set (ANOVA, *p* < 0.05).

All of the obtained and selected features from the untargeted approach then underwent a univariate analysis (fdr < 0.05) to understand how the different compound concentrations (log_10_-relative intensities) changed depending on the extraction types. Because of their occurrence and importance as polyphenols and methylxanthines contained in CBS, five specific identified compounds were selected among all of the obtained ion boxplots: catechin, epicatechin, procyanidin B1 (PCB1), and the methylxanthines theobromine and caffeine (Figure 3).

From these plots, it can be observed that the concentration of both flavan-3-ols (catechin and epicatechin) decreased after gastrointestinal digestion in comparison with that of the other extraction types. This has been previously reported by several authors and is mainly explained by the instability of these compounds in neutral or mildly alkaline environments that typify the intestinal phase of digestion. Moreover, these compounds are known to strongly interact with digestive enzymes, which leads to polymerization and aggregate formation of the flavan-3-ol monomers [35]. In different types of chocolate and cocoa beverages, the bioaccessibility of catechin and epicatechin was found to be between 77 and 79% and between 73 and 91%, respectively [37], which is in accordance with the results observed in the plots. However, in the absence of a food matrix, the degradation of these compounds could be even more notable. This was the case for different CBS-based beverages [14] and different types of tea [38] in which flavan-3-ols were degraded by up to 90% after gastrointestinal digestion. This was explained as being due to the autooxidation of these compounds under alkaline conditions. In guarana seeds, which have been reported to have a very similar flavonoid composition to that of cocoa, the bioaccessibility of catechin monomers was found to be between 65 and 95% while that of dimers (procyanidins) ranged from 50% to up to 140% [39], similar to the values obtained in this work. According to our results, the PCB1 concentration was in fact higher for all samples after gastrointestinal digestion in comparison with the samples that underwent organic solvent extraction or non-digestion extraction, which is also in accordance with the TTC results obtained for the biscuit samples. This could be explained by the previously mentioned phenomenon in which catechin monomers suffer from polymerization during gastrointestinal digestion, increasing the concentrations of procyanidin dimers, such as PCB1, and polymers. Furthermore, this increase in PCB1 bioaccessibility could have been boosted by the presence of fats in biscuits which, according to Ortega et al. [36], would enhance the digestibility of some cocoa compounds, especially procyanidins, under the alkaline conditions of intestinal digestion. However, the lower intensities of the digested samples in the catechin and epicatechin boxplots contradict the results for the total flavonoid content, in which values increased considerably for the digested biscuit samples, while they decreased for the samples containing the CBS ingredient alone. A similar scenario was found by Pešić et al. [35], who justified the higher-than-expected TFC values by the influence of phenolic acids on the overall determination of this parameter via the employed methodology.

For the two methylxanthines, theobromine and caffeine, a similar trend was found in which their intensities in the digested samples slightly decreased in comparison with the other two extractions, which had very similar intensities. Methylxanthines have been reported to be stable under gastric and intestinal conditions since they are not degraded by the pH conditions or enzymes [14].

### 3.5. In Vitro Intestinal Permeability of Bioactive Compounds through Caco-2 Cell Monolayers

After conducting the bioaccessibility studies, besides understanding how much of the bioactive compounds contained in the different samples were available for absorption after gastrointestinal digestion, we also determined the concentration that was actually able to permeate through the intestinal wall and pass into the bloodstream. This factor is of great importance since several types of biofunctionality have been attributed to the cocoa bean shell in vitro [3], but in order to allow for these processes to really happen in the human organism, it is mandatory for the compounds of interest to reach the bloodstream and, therefore, the target organs.

Transport through the small intestinal epithelium cells can occur through four different pathways—transporter-mediated, transcytosis, transcellular diffusion, and paracellular transport—and can be affected by different food components and substances. In this study, we employed Caco-2 cell monolayers to study intestinal absorption. Although these cells originated in colon tissue, they are known to create epithelial monolayers and mimic several functions characteristic of the small intestine, including differentiation with microvilli and formation of junctional structures, and the expression of different enzymes [40]. To increase the accuracy and mimic the natural process, only the digested extracts were tested for their absorption through Caco-2 cells. Prior to transport studies and in order to assure the integrity of the Caco-2 cell monolayer, the cell viability was tested after 2 and 4 h of cultivation in the presence of the digested extracts. The results showed cell viability values higher than 80% for all extracts (Figure S2); therefore, it was considered that the incubation conditions employed in the absorption studies were safe for Caco-2 cells. Additionally, the TEER measurements and the Lucifer Yellow assay confirmed the integrity of the monolayer after the transport experiments. Then, the transport of five representative target compounds contained in the samples (catechin, epicatechin, PCB1, caffeine, and theobromine) from the apical to the basolateral compartment of Caco-2 monolayers cultivated onto porous membranes was monitorized at T_0_ (initial concentration at the AP), T_1_ (concentration at the BL after 60 min), and T_2_ (concentration at the BL after 120 min). The apparent permeability coefficients (P_app_) resulting from the obtained data are shown in Table 1. In addition, the concentrations that were used for the P_app_ calculation and the absorption percentages at different times are presented in Tables S3 and S4, respectively.

Concerning flavan-3-ols, the apparent permeability coefficients for catechin and epicatechin ranged between 3.28 and 30.47 × 10^−6^ cm/s. These values are slightly high compared with other coefficients found in the literature. Deprez et al. [41] found an apparent permeability for catechin of 0.8 × 10^−6^ cm/s, while Fang et al. [42] found variable apparent permeabilities ranging from 0.29 to 33.90 × 10^−6^ cm/s for different flavonoids. Kosińska et al. found that, in purified extracts, catechin and epicatechin showed P_app_ values of 4.19 × 10^−6^ cm/s and 3.38 × 10^−6^ cm/s, respectively. Nevertheless, as stated by Mendes et al. [39], the considerable variability of catechin P_app_ coefficients reported in the literature can be explained by the complex phenotype of Caco-2 cells, which features different TEER values and levels of efflux transporter expression depending on the source and culture conditions. Furthermore, the paracellular permeability can vary significantly depending on the calcium concentration in the digestion cocktail, since it is believed that calcium ions contribute to the integrity of tight junctions and that their removal could lead to the enlargement of the intercellular spaces, with a consequent increase in P_app_ [41]. Indeed, several authors have hypothesized that paracellular transport is the preferential mechanism of intestinal absorption for polyphenols because of their high hydrophilicity and abundance of hydroxyl groups [21,43]. However, in studies where P_app_ values were lower, compound standards or purified extracts were tested. Indeed, it has been demonstrated that the different substances and compounds included in the food matrices can significantly increase the apparent permeability of flavan-3-ols. Some food components, such as proteins, are believed to protect catechins against oxidation by the culture medium during incubation on the apical side [43], while other components, such as capric or lauric acid, which have been reported to be present in CBS [3], could contribute to increased paracellular intestinal permeability due to tight junction opening [43]. In addition, it has been suggested that the presence of other cocoa polyphenols that may not be absorbed could affect tight junction functioning, enlarging the paracellular space [21], or inhibit intestinal ABC transporters (p-glycoprotein, MRP2, BCRP) that are responsible for bioactive compound efflux from the basolateral to the apical side [44]. Indeed, authors who tested flavanol standard solution absorption stated that the apparent permeability was low because basolateral-apical reflux was considerable [45]. Furthermore, several studies support the idea that co-transport of catechins with other components such as glycine and serine (both present in CBS as free aminoacids at percentages of 0.00973% and 0.01050%, respectively [46]) can enhance catechin absorption by regulating efflux transporters [47]. Moreover, theobromine and caffeine, which were present in considerable concentrations in our samples, have been demonstrated to increase the in vivo absorption of epicatechins since they are phosphodiesterase inhibitors that can prevent the inactivation of cAMP and cGMP, which are supposed to regulate MRP2 sorting [48]. All of these reasons could explain the uncommonly high values of P_app_ for catechin and epicatechin in our samples when compared with values reported by other studies where the target compounds were studied alone.

Considering the obtained catechin and epicatechin P_app_ coefficients, significant differences were found between samples, even between those with similar CBS percentages. This could be because, while the initial concentrations at T_0_ (Table S3) were in accordance with what was observed previously (generally higher for 20%-substituted samples and higher in digested biscuits compared with samples of the digested CBS ingredient alone), concentrations at T_1_ and T_2_ were similar for all samples. This led to very different P_app_ values and, unexpectedly, the samples with the highest CBS percentages gave the lowest P_app_ values. It was then hypothesized that, for these compounds, a maximum absorption rate in Caco-2 cells might occur, leading to an absorption plateau in which higher concentrations at T_0_ were not correlated with higher absorption rates. Thus, there was a limit to the extent to which these two flavan-3-ols could be absorbed. This absorption limit has been previously reported in vivo by other researchers for other flavan-3-ols such as (epi)gallocatechin [49]. Similarly, Gómez-Juaristi et al. [50] found that the availability of flavanols after the intake of different cocoa products was limited to 35% regardless of the initial dose. Nevertheless, the percentages of permeability obtained (Table S4) were in accordance with 2–15% catechin absorption for different types of tea [38], and those observed after 120 min were close to 25.3–29.8% absorption of epicatechin from chocolate and cocoa in human subjects, as reported by Baba et al. [51].

PCB1 was not detected on the basolateral side during our experiments, although it was present in detectable concentrations in the apical side. It is, therefore, concluded that this catechin–epicatechin dimer was not able to pass through the intestinal barrier, probably due to its bigger size and higher hydrophilicity. This has been frequently reported in the literature, although some authors have detected procyanidins at the BL after 5 h of incubation [39]. However, it is well known that procyanidins can be absorbed and accumulate in Caco-2 cells thanks to their affinity with the intestinal mucosa and luminal proteins. These findings were also observed for catechin monomers, although to a lesser extent [41]. Nevertheless, their accumulation and presence in the intestinal tract is of great importance, since these compounds can act at a local level to protect the intestinal mucosa against different types of damage by acting as antioxidant agents and exerting anti-inflammatory activities, as already reported by Rossin et al. [52]. In this way, they can decrease cancer risk, control cholesterol uptake, and inhibit glucose degradation enzymes [3].

Contrary to polyphenols, methylxanthines are believed to be absorbed by transcellular transport mediated through passive diffusion since their transport is linear over time, independent of transport direction and pH, and they have non-saturable (first-order) kinetics and high P_app_ coefficients [53]. In our study, both methylxanthines showed high P_app_ coefficients, which correlated well with the different samples. Significantly higher values were found for the P_app_ values of both caffeine and theobromine in the biscuit samples when compared with the samples containing the CBS ingredient alone. There was, therefore, an effect of the biscuit food matrix that led to increases in the absorption of both caffeine and theobromine through the Caco-2 cell monolayer. Methylxanthines could have bound to some biscuit food matrix proteins, which facilitated their passage through the cells. Monente et al. [54] found that 36.5% of the caffeine from digested spent coffee ground extracts was absorbed through Caco-2 cells after one hour of incubation, which is in accordance with the values of 32.43% and 35.48% obtained for CBS10 and CBS20 (Table S4) but lower than the values obtained for the biscuits after 1 h of incubation (45.77–48.91%) where the biscuit food matrix was present, in contrast with samples containing the CBS ingredient alone or the spent coffee ground extract. Regarding the P_app_ values obtained for caffeine (42.22–69.53 × 10^6^ cm/s), these are in accordance with those reported in other research articles, such as 46.3–53.5 × 10^6^ cm/s found by Smetanova et al. [53] for caffeine standards or 44.8 × 10^6^ cm/s found for guarana seeds [39]. Taking into account the fact that the U.S. Food and Drug Administration (FDA) has suggested that caffeine is a model drug for in vitro permeability assay validation [47], these values could also serve as evidence for the validation of the P_app_ values obtained for the other target compounds.

Concerning methylxanthines in cocoa, theobromine in particular, the information in the literature about in vitro absorption is limited. However, in vivo studies have demonstrated that cocoa methylxanthines are highly bioavailable, with maximum absorption after 30–60 min for caffeine and 2–2.5 h for theobromine [55,56]. Our results agree with these data, since higher permeability percentages were found for caffeine in comparison with theobromine at times 1 and 2, suggesting a faster absorption rate. On the other hand, theobromine absorption percentage values seemed to be still increasing after 2 h of incubation, which suggests that the absorption peak had not been reached. Indeed, theobromine was the only studied compound where values at T_2_ were found to be double those at T_1_ (T_2_ values were 1.94–2.12-fold greater than T_1_ values for theobromine, while they were 1.63–1.78, 1.56–1.86, and 1.47–1.65-fold greater for catechin, epicatechin, and caffeine, respectively).

## 4. Conclusions

The present study, which is a continuation of a previous one [11], highlights the potential of using CBS as a functional ingredient for high-fiber functional biscuits, with the possibility of adapting this product for diabetic consumers through the addition of tagatose. It was demonstrated that the substitution of sucrose with tagatose as a sweetener did not affect the bioavailability of bioactive compounds contained in the CBS-based biscuits. The results highlight the importance of considering the food matrix when studying polyphenol bioaccessibility. In this study, it was observed that, although the food matrix could retain bioactive compounds by binding them with the food components, it also has a protection effect, preventing bioactive compounds from degradation during gastrointestinal digestion. Furthermore, a compound liberation effect was observed during in vitro digestion of the biscuits, which increased the bioaccessibility of the bioactive compound and gave a considerable α-glucosidase inhibition capacity, which confirms their anti-diabetic potential. CBS bioactive compounds have been shown to permeate the intestinal barrier after in vitro digestion of functional biscuits. The exception is the procyanidin B1, which can still exert different functionalities at the local intestine level. The absorption of CBS methylxanthines was high, it correlated well with the results of previously reported in vivo studies, and it seemed to be boosted by the food matrix presence. To our knowledge, this is the first study to report the in vitro absorption of CBS methylxanthines. CBS has been proven to be an optimal revalorized ingredient for functional biscuits, and its biofunctional potential has been demonstrated.

## Figures and Tables

**Table 1:** Apparent permeability coefficients (P_app_) of the five target compounds found for the digested samples of CBS biscuits and CBS ingredient alone at different concentrations. Statistical comparisons were made using one-way ANOVA and Duncan’s post-hoc test at 95% confidence.

	P_app_ × 10^6^ (cm/s)												
	Catechin			Epicatechin			PCB1	Caffeine			Theobromine		
CBS10	30.47	±	3.64 ^a^	8.89	±	0.39 ^c^	n.d.	42.22	±	0.91 ^c^	41.89	±	0.61 ^d^
CBS20	22.83	±	1.07 ^bc^	4.68	±	0.13 ^d^	n.d.	45.00	±	2.13 ^c^	49.14	±	0.48 ^c^
S10	25.35	±	2.84 ^b^	15.92	±	0.69 ^a^	n.d.	69.53	±	0.42 ^a^	58.84	±	0.58 ^b^
S20	9.05	±	0.60 ^d^	3.28	±	0.15 ^e^	n.d.	62.46	±	0.36 ^b^	61.02	±	1.21 ^b^
T10	19.73	±	2.10 ^c^	11.33	±	0.32 ^b^	n.d.	66.66	±	4.47 ^a^	58.80	±	2.35 ^b^
T20	20.88	±	3.10 ^bc^	4.44	±	0.36 ^d^	n.d.	61.99	±	2.67 ^b^	65.80	±	1.80 ^a^

Means followed by different superindexes indicate significant differences at *p* < 0.05 for the same compound among the six different samples; data are expressed as mean values (*n* = 3) ± standard deviations.

## Supplementary Materials

The following are available online at https://www.mdpi.com/2076-3921/9/11/1164/s1, Table S1: W4M, steps and parameters, Table S2: Total phenolic content, total flavonoid content, total tannin content, antioxidant capacity, and α-glucosidase inhibition capacity (numerical values), Figure S1: Supplementary information to the score plots for the multivariate modeling using PCA or PLS-DA, Figure S2: Cell viability in Caco-2 cells, Table S3: Concentrations at the initial time (T0, apical side) and after 60 and 120 min (T1 and T2, basolateral side), Table S4: Percentage of permeated compound on the basolateral side after 60 and 120 min (T1 and T2, basolateral side) in relation to the concentration at T0 on the apical side.
