# Supplementary material for: Polyphenolic and Methylxanthine Bioaccessibility of Cocoa Bean Shell Functional Biscuits: Metabolomics Approach and Intestinal Permeability through Caco-2 Cell Models
Source: Antioxidants (Basel). 2020 Nov 22;9(11):1164. doi: 10.3390/antiox9111164 (PMC7700373; doi:10.3390/antiox9111164)
Supplement: Supplementary file 1 [file antioxidants-09-01164-s001.pdf]

antioxidants

MDPI

Article Supplementary Material

Article

Polyphenolic and Methylxanthine Bioaccessibility of Cocoa Bean Shell Functional Biscuits: Metabolomics Approach and Intestinal Permeability through Caco-2 Cell Models

Olga Rojo-Poveda[1,2,*], Letricia Barbosa-Pereira[2,3], Charaf El Khattabi[4], Estelle N.H. Youl[4,5], Marta Bertolino[2], Cédric Delporte[1,6], Stéphanie Pochet[4] and Caroline Stévigny[1]

[1] RD3 Department-Unit of Pharmacognosy, Bioanalysis and Drug Discovery, Faculty of Pharmacy, Université libre de Bruxelles, 1050 Brussels, Belgium; Cedric.Delporte@ulb.be (C.D.); Caroline.Stevigny@ulb.be (C.S.)

[2] Department of Agriculture, Forestry and Food Sciences (DISAFA), University of Turin, 10095 Grugliasco, Italy; letricia.barbosa.pereira@usc.es (L.B.-P.); marta.bertolino@unito.it (M.B.)

[3] Department of Analytical Chemistry, Nutrition and Food Science, Faculty of Pharmacy, University of Santiago de Compostela, 15782 Santiago de Compostela, Spain

[4] Laboratory of Pharmacology, Pharmacotherapy and Pharmaceutical care, Université libre de Bruxelles, 1050 Brussels, Belgium; Charaf.El.Khattabi@ulb.ac.be (C.E.K.); yestella@yahoo.fr (E.N.H.Y.); Stephanie.Pochet@ulb.ac.be (S.P.)

[5] Laboratory of drug development, Faculty of Medicine and Pharmacy, Université Joseph Ki-Zerbo, BP 958 Ouagadougou 09, Burkina Faso

[6] Analytical Platform of the Faculty of Pharmacy (APFP), Faculty of Pharmacy, Université libre de Bruxelles, 1050 Brussels, Belgium

* Correspondence: Olga.Rojo.Poveda@ulb.be

Received: date; Accepted: date; Published: date

Abstract: Cocoa bean shell (CBS), a by-product with considerable concentrations of bioactive compounds and proven biofunctional potential, has been demonstrated to be a suitable ingredient for high-fiber functional biscuits adapted to diabetic consumers. In this work, the in vitro bioaccessibility and intestinal absorption of polyphenols and methylxanthines contained in these biscuits were evaluated, and the effect of the food matrix was studied. Biscuits containing CBS and the CBS alone underwent in vitro digestion followed by an intestinal permeability study. The results confirmed that compounds were less bioavailable in the presence of a food matrix, although the digestion contributed to their release from this matrix, increasing the concentrations available at the intestinal level and making them capable of promoting antioxidant and antidiabetic activities. After digestion, CBS biscuits were shown to possess α-glucosidase inhibition capacity comparable to that of acarbose. Moreover, the presence of the food matrix improved the stability of polyphenols throughout the digestion process. Intestinal absorption of flavan-3-ols seemed to be limited to a maximum threshold and was therefore independent of the sample, while procyanidin was not absorbed. Methylxanthine absorption was high and was boosted by the presence of the food matrix. The results confirmed the biofunctional potential of CBS-based biscuits.

Keywords: cocoa bean shell; biscuits; functional foods; polyphenols; methylxanthines; in vitro digestion; α-glucosidase inhibition; bioaccessibility; metabolomics; Caco-2 absorption

Table S1. Steps and parameters employed for the metabolomics analysis in the W4M platform (https://workflow4metabolomics.usegalaxy.fr/). Adapted from the work of Souard et al. [19]

**xcms.xcmsSet** Filtration and Peak Identification using xcmsSet function from xcms R package to preprocess LC/MS data for relative quantification and statistical analysis (Galaxy Version 3.6.1+galaxy1)	
Scan range option	*hide*
Extraction method for peaks detection	*centWave*
Max tolerated ppm *m/z* deviation in consecutive scans in ppm	*15*
Min,Max peak width in seconds	*20,60*
Signal/Noise threshold	*10*
Min *m/z* difference	*0.01*
Peak limits method	*Peak limits are found through descent on the mexican hat filtered data*
Prefilter step for the first phase	*3,1500*
Noise filter	*500*

**xcms.group** Group peaks together across samples using overlapping *m/z* bins and calculation of smoothed peak distributions in chromatographic time. (Galaxy Version 3.6.1+galaxy1)	
Method to use for grouping	*density*
Bandwidth	*10*
Minimum fraction of samples necessary	*0.1*
Width of overlapping *m/z* slices	*0.025*
Maximum number of groups to identify in a single *m/z* slice	*50*

**xcms.retcor** Retention Time Correction using retcor function from xcms R package (Galaxy Version 3.6.1+galaxy1)	
Method to use for retention time correction	*peakgroups*
Smooth method	*loess*
Number of extra peaks to allow in retention time correction correction groups	*1*
Number of missing samples to allow in retention time correction groups	*100*
Degree of smoothing for local polynomial regression fitting	*0.2*
Family	*gaussian*
Plottype	*deviation*

**xcms.group** Group peaks together across samples using overlapping *m/z* bins and calculation of smoothed peak distributions in chromatographic time. (Galaxy Version 3.6.1+galaxy1)	
Method to use for grouping	*density*
Bandwidth	*5*
Minimum fraction of samples necessary	*0.1*
Width of overlapping *m/z* slices	*0.025*
Maximum number of groups to identify in a single m/z slice	*50*

**xcms.fillPeaks** Integrate the signal in the region of that peak group not represented and create a new peak (Galaxy Version 3.6.1+galaxy0)	
Filling method	*chrom*

**Intensity Check** Calculates, among other metrics, the relative intensity of some sample group features with respect to the remaining samples (Galaxy Version 1.2.8)	
Computation method	*Between one class and all the remaining samples*
Class column	*Subclass1_SampleSubGroup*
Selected class	*blank*
Calculate the mean fold change	*Yes*
Where should the class be placed for the mean fold change calculation?	*Denominator*

**Generic_Filter** Removes elements according to numerical or qualitative values (Galaxy Version 2020.01)	
**Deleting samples and/or variables according to Numerical values**	*Yes*
Identify the parameter to filter	*Variable metadata*
Name of the column to filter	*fold_Other_VS_blank*
Interval of values to remove	*lower*
Remove all values lower than	*2.5*
**Deleting samples and/or variables according to Qualitative values**	*Yes*
Removing a level in factor	*Sample metadata*
Name of the column to filter	*sampleType*
Remove factor when	*blank*

**Normalization** of preprocessed data (Galaxy Version 1.0.7)	
Normalization method	*Quantitative Variable*
Name of the column	*WeightProportion*

**Intensity Check** Calculates, among other metrics, the relative intensity of some sample group features with respect to the remaining samples (Galaxy Version 1.2.8)	
Computation method	*Between one class and all the remaining samples*
Class column	*Subclass1_SampleSubGroup*
Selected class	*H2O (Digestion blank)*
Calculate the mean fold change	*Yes*
Where should the class be placed for the mean fold change calculation?	*Denominator*

**Generic_Filter** Removes elements according to numerical or qualitative values (Galaxy Version 2020.01)	
**Deleting samples and/or variables according to Numerical values**	*Yes*

Identify the parameter to filter	*Variable metadata*
Name of the column to filter	*fold_Other_VS_H2O*
Interval of values to remove	*lower*
Remove all values lower than	*1.0*
**Deleting samples and/or variables according to Qualitative values**	*Yes*
Removing a level in factor	*Sample metadata*
Name of the column to filter	*Subclass1_SampleSubGroup*
Remove factor when	*blank*

**Batch_correction** Corrects intensities for signal drift and batch-effects (Galaxy Version 2.1.2)	
Type of regression model	*linear*

**Transformation** Transforms the dataMatrix intensity values (Galaxy Version 2.2.0)	
Method	*Log10*

**Quality Metrics** Metrics and graphics to check the quality of the data (Galaxy Version 2.2.6)	
Coefficient of Variation	*Yes*
Which type of CV calculation should be done	*Only pool CV*
Threshold	*0.3*
Advanced parameters	*Use default*

**Generic_Filter** Removes elements according to numerical or qualitative values (Galaxy Version 2020.01)	
**Deleting samples and/or variables according to Numerical values**	*Yes*
Identify the parameter to filter	*Variable metadata*
Name of the column to filter	*Pool_CV*
Interval of values to remove	*upper*
Remove all values upper than	*0.3*
**Deleting samples and/or variables according to Qualitative values**	*Yes*
Removing a level in factor	*Sample metadata*
Name of the column to filter	*sampleType*
Remove factor when	*pool*

**Multivariate** PCA, PLS and OPLS (Galaxy Version 2.3.10)	
Y Response (for (O)PLS(-DA) only)	*1) PCA: keep the default (none); 2) (O)PLS(-DA): indicate the name of the column of the sample table to be modeled* ***(➔ "Subclass2_ExtractionType")***
Number of predictive components	*1) PCA and PLS(-DA): 4*
Number of orthogonal components (for OPLS(-DA) only)	*Notes: 1) PCA and PLS(-DA): 0*
Samples to be tested	*no*
Advanced graphical parameters	*Full parameter list*

Graphic type	*summary*
Ellipses	***"Subclass2_ExtractionType"***
Sample labels	***"Subclass2_ExtractionType"***
Component to be displayed as abscissa	***1***
Component to be displayed as ordinate	***2***
Amount by which plotting text should be magnified relative to the default	*0.4*
Advanced computational parameters	*Full parameter list*
Scaling	*Standard*
Permutation testing for (O)PLS(-DA): Number of permutations	*20*
Log10 transformation	*No (➔ already done in the transformation step)*
Algorithm	*Default*
Number of cross-validation segments	*7*

**Univariate** Univariate statistics (Galaxy Version 2.2.4)	
Factor of interest	***"Subclass2_ExtractionType"***
Test	*Analysis of variance (qualitative, more than 2 levels)*
Method for multiple testing correction	*fdr*
(Corrected) p-value significance threshold	*0.05*

Table S2. Total phenolic content, total flavonoid content, total tannin content, antioxidant capacity, and α-glucosidase inhibition capacity for the three extraction types (Org, Dig, and No_Dig) performed for the CBS biscuits (S10, T10, S20 and T20) and CBS powder alone without food matrix (CBS10 and CBS20). Results are presented as mean ± standard deviation (n=6).

**TPC (mg GAE/g)**	Organic extraction	In vitro digestion	No-digestion
S10	0.21 ± 0.04	0.26 ± 0.15	0.16 ± 0.07
T10	0.25 ± 0.09	0.50 ± 0.14	0.13 ± 0.02
CBS10	1.32 ± 0.12	0.77 ± 0.10	0.87 ± 0.11
S20	0.43 ± 0.05	0.47 ± 0.26	0.41 ± 0.03
T20	0.54 ± 0.13	0.59 ± 0.09	0.49 ± 0.11
CBS20	2.73 ± 0.35	1.50 ± 0.19	1.68 ± 0.22

**TFC (mg CE/g)**	Organic extraction	In vitro digestion	No-digestion
S10	0.15 ± 0.01	0.25 ± 0.06	0.11 ± 0.02
T10	0.13 ± 0.01	0.18 ± 0.06	0.10 ± 0.01
CBS10	0.29 ± 0.03	0.19 ± 0.01	0.24 ± 0.02
S20	0.35 ± 0.04	0.44 ± 0.10	0.17 ± 0.03
T20	0.27 ± 0.03	0.31 ± 0.04	0.18 ± 0.06
CBS20	0.61 ± 0.06	0.38 ± 0.03	0.47 ± 0.04

**TTC (mg CE/g)**	Organic extraction	In vitro digestion	No-digestion
S10	0.03 ± 0.00	0.05 ± 0.03	0.02 ± 0.01
T10	0.02 ± 0.01	0.03 ± 0.01	0.02 ± 0.01
CBS10	0.22 ± 0.03	0.07 ± 0.00	0.07 ± 0.00
S20	0.06 ± 0.01	0.10 ± 0.06	0.05 ± 0.01
T20	0.04 ± 0.02	0.06 ± 0.03	0.03 ± 0.01
CBS20	0.45 ± 0.09	0.13 ± 0.01	0.14 ± 0.01

**Antioxidant capacity (μmol TE/g)**	Organic extraction	In vitro digestion	No-digestion
S10	1.31 ± 0.25	1.78 ± 0.16	0.86 ± 0.09
T10	1.02 ± 0.56	1.30 ± 0.46	0.64 ± 0.29
CBS10	3.93 ± 0.37	2.83 ± 0.20	3.51 ± 0.23
S20	3.17 ± 0.13	3.40 ± 0.70	1.86 ± 0.15
T20	2.78 ± 0.43	3.24 ± 0.38	1.33 ± 0.43
CBS20	8.23 ± 0.84	5.49 ± 0.38	6.80 ± 0.44

**α-glu inhibition capacity (μmol AcE/g)**	Organic extraction	In vitro digestion	No-digestion
S10	1.05 ± 0.24	1.66 ± 0.39	0.10 ± 0.03
T10	1.15 ± 0.30	1.56 ± 0.86	0.15 ± 0.08
CBS10	1.39 ± 0.30	0.63 ± 0.14	0.95 ± 0.21
S20	2.88 ± 0.44	5.04 ± 0.60	0.20 ± 0.05
T20	2.18 ± 0.38	5.65 ± 0.80	0.22 ± 0.09
CBS20	2.87 ± 0.68	1.22 ± 0.27	1.84 ± 0.41

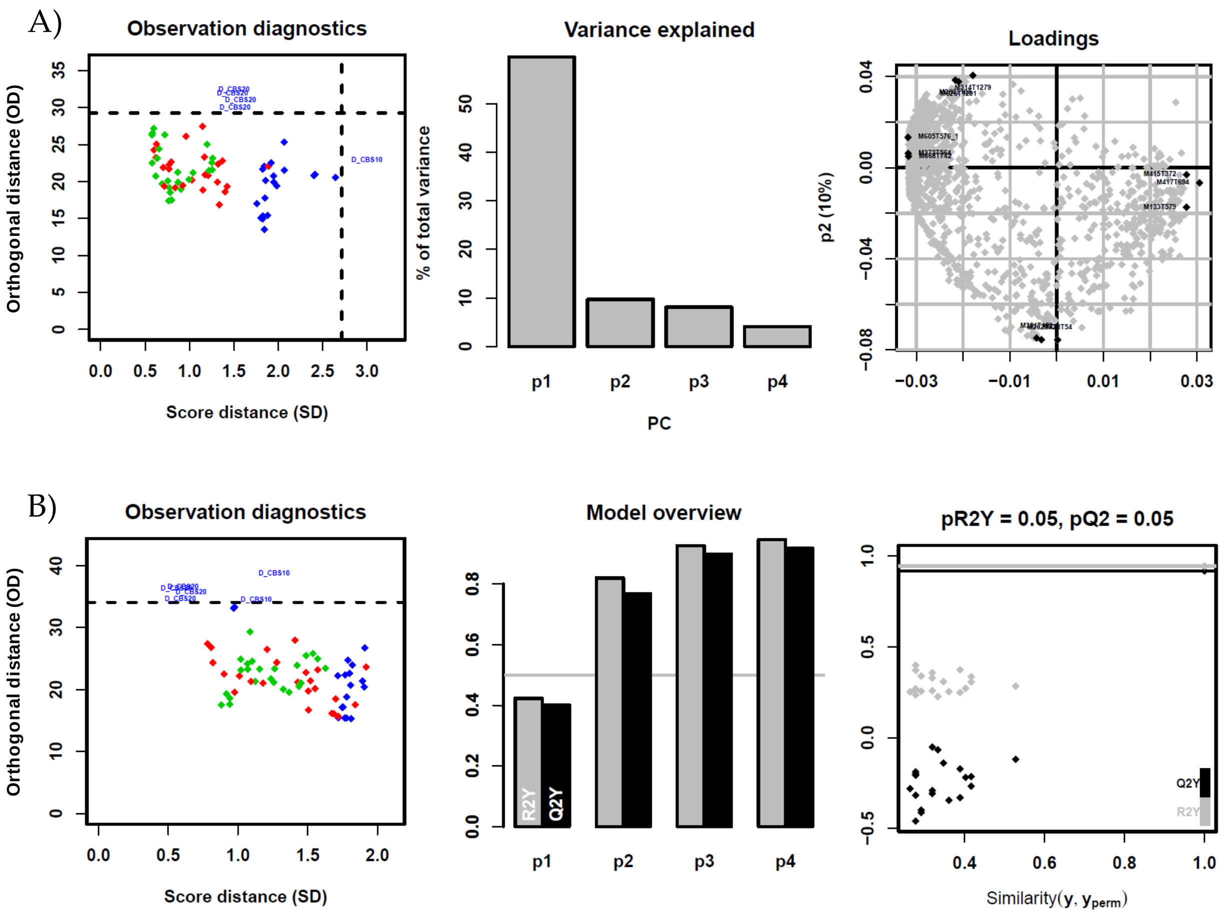

Figure S1. Supplementary information to the score plots for the multivariate modeling using PCA (**A**) or PLS-DA (**B**). The observation diagnostic plot shows the distances within and orthogonal to the selected score plane. For PCA, the variance explained by the first four principal components is shown and, on the loading plots, the names of the 6 variables with most extreme values in each direction, is indicated. For PLS modeling, an additional diagnostic plot shows the Q2Y (and R2Y) values from the model (horizontal lines) compared to the values from the models obtained after random permutations of the y response (dots) [19].

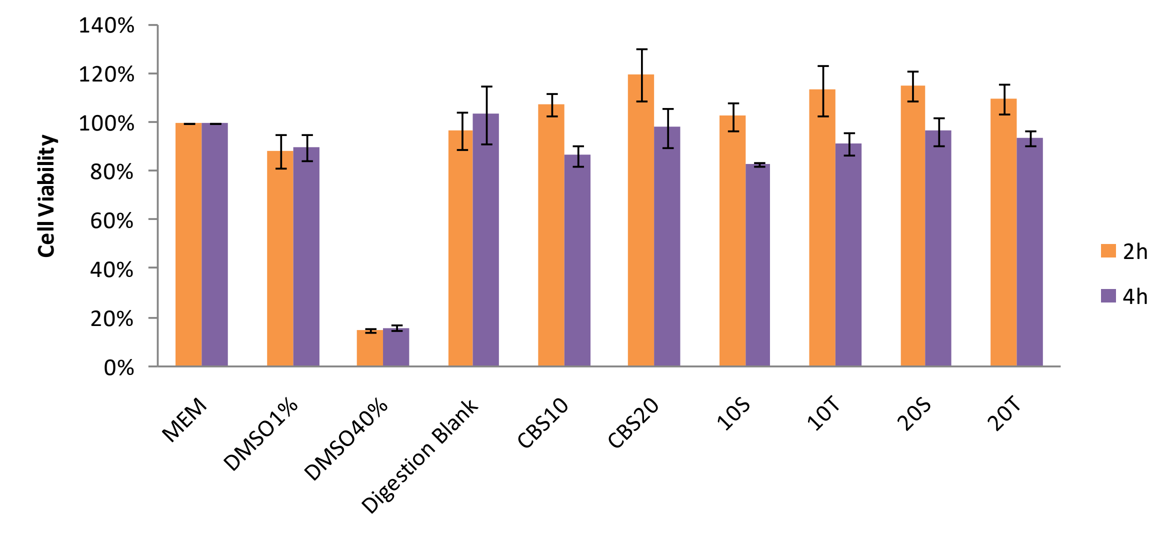

Figure S2. Cell viability in Caco-2 cells of the culture medium used as positive control (MEM), the DMSO percentage used for sample solubilization (DMSO 1%), high-DMSO concentration used as negative control (DMSO 40%), the digestion cocktail (Digestion blank), the digested CBS ingredient alone at different concentrations (CBS10 and CBS20), and the biscuits developed with sugar or tagatose and different percentages of CBS (S10, T10, S20, and T20). Results are presented as mean ± standard error of the mean (SEM) ($n \geq 5$).

Table S3. Concentrations at the initial time (T_0, apical side) and after 60 and 120 minutes (T_1 and T_2, basolateral side) of the five target compounds for the digested samples of the CBS ingredient alone and biscuits with different CBS-added concentrations.

	Concentration T_0 (μg/g)				
	Catechin	Epicatechin	PCB1	Caffeine	Theobromine
CBS10	0.39 ± 0.03	0.89 ± 0.04	0.46 ± 0.10	48.10 ± 0.95	129.69 ± 3.48
CBS20	0.54 ± 0.03	1.72 ± 0.05	0.68 ± 0.14	82.84 ± 2.73	198.53 ± 2.79
S10	0.48 ± 0.09	0.50 ± 0.05	0.34 ± 0.06	35.67 ± 0.88	121.81 ± 2.58
S20	1.26 ± 0.09	2.23 ± 0.09	0.89 ± 0.07	65.76 ± 0.40	180.92 ± 3.80
T10	0.56 ± 0.05	0.63 ± 0.02	0.38 ± 0.15	40.83 ± 1.14	147.18 ± 1.34
T20	0.57 ± 0.07	1.72 ± 0.06	0.38 ± 0.09	83.14 ± 1.16	203.16 ± 1.21

	Concentration T_1 (μg/g)				
	Catechin	Epicatechin	PCB1	Caffeine	Theobromine
CBS10	0.08 ± 0.00	0.05 ± 0.00	n.d.	15.59 ± 0.37	31.64 ± 2.61
CBS20	0.08 ± 0.01	0.06 ± 0.00	n.d.	29.36 ± 1.13	58.24 ± 2.40
S10	0.09 ± 0.01	0.06 ± 0.01	n.d.	17.45 ± 0.57	38.84 ± 2.42
S20	0.08 ± 0.00	0.05 ± 0.00	n.d.	30.10 ± 0.90	61.41 ± 4.83
T10	0.08 ± 0.00	0.05 ± 0.00	n.d.	19.61 ± 1.27	47.41 ± 5.36
T20	0.08 ± 0.01	0.05 ± 0.01	n.d.	38.00 ± 0.50	74.85 ± 5.03

	Concentration T_2 (μg/g)				
	Catechin	Epicatechin	PCB1	Caffeine	Theobromine
CBS10	0.14 ± 0.00	0.08 ± 0.00	n.d.	26.73 ± 0.23	54.24 ± 1.03
CBS20	0.14 ± 0.00	0.10 ± 0.00	n.d.	50.33 ± 0.61	99.84 ± 2.23
S10	0.14 ± 0.01	0.10 ± 0.01	n.d.	29.91 ± 0.55	66.58 ± 2.53
S20	0.13 ± 0.00	0.09 ± 0.00	n.d.	51.60 ± 0.53	105.27 ± 0.71
T10	0.13 ± 0.00	0.08 ± 0.00	n.d.	33.61 ± 1.43	81.27 ± 4.36
T20	0.14 ± 0.01	0.09 ± 0.00	n.d.	65.14 ± 2.10	128.31 ± 4.50

Table S4. Percentage of permeated compound on the basolateral side after 60 and 120 minutes (T1 and T2, basolateral side) in relation to the concentration at T0 on the apical side of the five target compounds for the digested samples of the CBS ingredient alone and biscuits with different CBS–added concentrations.

		Permeability (%)				
		Catechin	Epicatechin	PCB1	Caffeine	Theobromine
CBS10	T1	19.82 ± 1.86	5.54 ± 0.29	n.d.	32.43 ± 1.27	24.37 ± 1.38
	T2	35.45 ± 4.23	10.34 ± 0.45	n.d.	49.12 ± 1.06	48.73 ± 0.71
CBS20	T1	15.35 ± 0.60	3.24 ± 0.09	n.d.	35.48 ± 2.12	29.33 ± 0.87
	T2	26.55 ± 1.25	5.44 ± 0.15	n.d.	52.35 ± 2.48	57.17 ± 0.56
S10	T1	18.08 ± 4.12	11.35 ± 1.80	n.d.	48.91 ± 0.90	31.87 ± 1.31
	T2	29.49 ± 3.30	18.52 ± 0.81	n.d.	80.89 ± 0.49	68.45 ± 0.68
S20	T1	6.33 ± 0.54	2.43 ± 0.28	n.d.	45.77 ± 1.34	33.92 ± 1.97
	T2	10.53 ± 0.70	3.81 ± 0.18	n.d.	72.66 ± 0.41	70.98 ± 1.41
T10	T1	13.70 ± 1.20	7.81 ± 0.28	n.d.	48.08 ± 3.91	32.21 ± 3.61
	T2	22.96 ± 2.44	13.18 ± 0.38	n.d.	77.54 ± 5.20	68.40 ± 2.73
T20	T1	14.52 ± 1.09	3.13 ± 0.44	n.d.	45.72 ± 1.25	36.83 ± 2.26
	T2	24.29 ± 3.60	5.16 ± 0.42	n.d.	72.11 ± 3.11	76.55 ± 2.09

© 2020 by the authors. Submitted for possible open access publication under the terms and conditions of the Creative Commons Attribution (CC BY) license (http://creativecommons.org/licenses/by/4.0/).
